# Genetic diversity and phylogenetic relationships of *Calotes* and *Uromastyx* in the Cholistan Desert, Pakistan, based on *COI* gene analysis

**DOI:** 10.1371/journal.pone.0324053

**Published:** 2025-06-17

**Authors:** Javed Hussain, Gulnaz Afzal, Muhammad Zeshan Haider, Iram Qadeer, Shazia Perveen, Hafiz Ishfaq Ahmad, Ahmed A. El-Mansi, Abdelalim A. Gadallah, Kasim Sakran Abass

**Affiliations:** 1 Department of Zoology, The Islamia University of Bahawalpur, Pakistan; 2 Department of Zoology, The Govt. Sadiq College Women University, Bahawalpur, Pakistan; 3 Department of Zoology, The Women University Multan, Mattital Campus, Multan, Pakistan; 4 Department of Animal Breeding and Genetics, Faculty of Veterinary and Animal Sciences, The Islamia University of Bahawalpur, Bahawalpur, Pakistan; 5 Biology Department, Faculty of Science, King Khalid University, Abha, Saudi Arabia; 6 Biology Department, College of Science, Jazan University, Jazan, Kingdom of Saudi Arabia; 7 Department of Physiology, Biochemistry, and Pharmacology, College of Veterinary Medicine, University of Kirkuk, Kirkuk, Iraq; Sunrise University, INDIA

## Abstract

The Cholistan Desert of Pakistan harbors unique reptile diversity, including ecologically significant agamid lizards of the genera *Calotes* and *Uromastyx*, yet their genetic structure remains poorly understood. This study presents the first mitochondrial DNA barcode assessment of these taxa in the region, analyzing 658 bp of the cytochrome c oxidase I (*COI*) gene from 19 specimens collected across seven desert sites. We employed a combination of distance-based (p-distance, K2P) and phylogenetic methods (Maximum Likelihood, Bayesian Inference) to evaluate genetic diversity and evolutionary relationships. Results revealed pronounced divergence between genera (24–30% K2P distance), with *Uromastyx* populations showing remarkably low intraspecific variation (0–1%), contrasting with higher diversity in *Calotes* (0–14%). Demographic analyses suggested stable populations (Tajima’s D = 0.93, p > 0.10), though haplotype networks indicated limited gene flow (Nm = 0.12). Our findings: (1) provide the first genetic baseline for these ecologically important desert lizards, (2) identify *Uromastyx* as potentially more vulnerable to genetic erosion, and (3) demonstrate the utility of *COI* barcoding for rapid biodiversity assessment in understudied arid ecosystems. The study highlights the Cholistan Desert as an evolutionary significant zone for agamid lizards while underscoring the need for integrated taxonomic approaches to address potential cryptic diversity. All sequence data are publicly available (GenBank PQ896696-PQ896705) to support future conservation genomic studies.

## Introduction

Cholistan is an arid desert region in the Province of Punjab, Pakistan that has a rich and diverse wildlife, especially reptiles who are threatened primarily by habitat degradation and predation. The diversty of herpetofauna in Pakistan is remarkable and includes 24 amphibian species and 195 reptiles; 13 reptiles are endemic [1,2]. Of these, the lizard is the foremost prominent group which composes families like Agamidae and Uromastycidae [3]. However, the conservation issue of reptiles is faced with some challenges this is evidenced by the International Union for Conservation of Nature (IUCN) Red List classification which reported that about 27.6% of reptiles are threatened with extinction risk and with many remaining unassessed Red List assessment [4].

The Cholistan Desert of Pakistan is a suitable habitat for a wide variety of reptilian species *Calotes* and *Uromastyx*. Molecular genetics of these lizards have not been well explored thus leaving large gaps on their phylogenetic tree and overall genetic variability. The genus *Calotes* is relatively frequent and these lizards occurs widely in the Oriental region: they are commonly found in plantation and garden environments where human interference is fairly common [5]. The genus *Calotes* is known for 34 species, where few of those are challenging for taxonomic classification and conservational biology [6,7].

*Uromastyx* (spiny-tailed lizard) on the other hand is however comprised of 17 species based on morphological identification. However, their phylogenetic analysis and classification is still ambiguous and continues to be an area of discussion [8,9]. *Uromastyx hardwickii* ranges from North Africa through the Indo-Pakistan subcontinent is particularly endangered due to threats as habitat loss and poaching the for the purpose of meat, skin and oil [10]. Yet, numerous kinds of the *Uromastyx* species are currently threatened by overexploitation, which includes documented export of more than 367,000 specimens between 1977 and 2005 [11]. Similarly, the genus *Calotes*—particularly the *C. versicolor* complex—has undergone repeated reassessments as molecular data uncover hidden diversity across South and Southeast Asia [5,12]. These studies collectively highlight the limitations of morphology-based taxonomy and underscore the necessity of genetic approaches for accurate biodiversity assessment.

Mitochondrial DNA markers, particularly the cytochrome c oxidase subunit I (*COI*) gene, have emerged as powerful tools for resolving such taxonomic uncertainties in reptiles [13]. The *COI* gene’s utility stems from its high mutation rate, which provides resolution at both species and population levels, as demonstrated in recent agamid studies [12,14]. This study provides the first *COI* barcode data for Cholistan’s *Calotes* and *Uromastyx*, addressing a key knowledge gap in Agamidae research. We document genetic diversity in threatened populations like *U. hardwickii* (historically overexploited) while demonstrating COI’s utility for rapid biodiversity assessment in arid ecosystems. Our findings establish both baseline genetic data and a framework for future genomic studies of these ecologically important desert lizards.

## Materials and methods

### Sample collection

A total of 19 individual lizard specimens were collected from seven selected sites in the Cholistan Desert (28°15′N to 29°45′N, 70°00′E to 72°00′E) between April and August 2024. Specimens were identified in the field based on morphological characteristics following standard taxonomic keys [5], with voucher specimens deposited at the Islamia University of Bahawalpur Zoological Museum (IUB-ZM2024–001–019). Blood and tissue samples were obtained from identified species of *Calotes* and *Uromastyx* using sterile techniques. Venous blood was obtained by using sterile hypodermic needles and syringes. The blood samples were preserved in Ethylenediamine tetraacetic acid (EDTA) blood collecting tubes and kept refrigerated at –20 °C to ensure DNA integrity before extraction. In addition, samples of the lizard tail samples were also collected and preserved as required for DNA extraction. After the study, the lizards were reintroduced into their original environment. This study was conducted following the approval of the research synopsis (Approval No. 1112/AS&R) and in strict adherence to the ethical guidelines for animal research as outlined by the Institutional Bio-Safety Committee (IBSC) of the Islamia University of Bahawalpur (Approval No. 455/ORIC). Throughout the research process, no procedures involving anesthesia, euthanasia, or animal sacrifice were employed. Furthermore, no instances of individual animal suffering were observed during the course of the study.

### DNA extraction

The extraction of DNA was done using Thermo Fischer Scientific Gene JET Genomic DNA Purification kit for TAQ DNA Polymerase, TAQ Buffer, dNTPs. For blood samples, the extraction protocol included enzymatic lysis using proteinase K, followed by purification through a spin-column method. In brief, 100 µL of whole blood was treated with 200 µL of lysis buffer and 20 µL of proteinase K and incubated at 56°C for 10 minutes. This process started with transferring of 20 µL of proteinase K into a microcentrifuge tube to which anticoagulated blood was added either 50–100 µL for non-nucleated blood or 5–10 µL for nucleated blood; the final volume each tube was topped up to 220 µL with PBS. The sample was then treated with 200 µL of Buffer AL at the temperature 56°C for 10 minutes. Two hundred microliters of ethanol were added and vortexed to get a homogeneous solution. The mixture recovered was then applied to DNeasy Mini spin column placed in a collection tube and centrifugation was done at 6000 x g (8000 rpm) for one minute. The spin column was then washed in Buffer AW1 and AW2 to remove the remaining contaminants followed by DNA samples were air-dried, and DNA was eluted with Buffer AE to get rid of contaminants and obtain DNA for further applications.

### Primer design

The mitochondrial cytochrome c oxidase subunit I (*COI*) gene was targeted for genetic diversity and phylogenetic analysis. The primers used for PCR amplification were as follows:

• **Forward Primer:** 5’ GGTCAACAAATCATAAAGATATTGG 3’
• **Reverse Primer:** 5’ TAAACTTCAGGGTGACCAAAAAATCA 3’

These primers enhanced amplification of target DNA sequence for further characterization.

### Polymerase chain reaction (PCR) and gene amplification

PCR was performed in a 25 µL reaction mixture containing 12.5 µL of 2X DreamTaq PCR Master Mix (Thermo Fisher Scientific), 1.0 µL (10 pmol) of each primer, 2.0 µL of template DNA (~50 ng), and 8.5 µL of nuclease-free water. Amplification was conducted using a Bio-Rad T100™ Thermal Cycler under the following conditions: initial denaturation at 95°C for 4 min, followed by 35 cycles of denaturation at 94°C for 35 sec, annealing at 65°C for 45 sec, and extension at 72°C for 90 sec, with a final extension at 72°C for 8 min. The amplification procedure was finished with a final extension step that lasted eight minutes at 72°C. To confirm effective amplification, the PCR products were seen on a 1.5% agarose gel stained with SYBR® Safe DNA and documented under UV light using a GelDoc™ XR+ system (Bio-Rad, USA). Out of 19, 10 lizard’ specimens were amplified and sequenced of *COI* gene. Amplified PCR products were sent for sequencing to verify the species identity and comprehend the genetic makeup of the lizard samples (*Calotes* and *Uromastyx*).

### DNA sequencing and data processing

Successful amplicons (~600–650 bp) were purified using the ExoSAP-IT™ PCR Product Cleanup Reagent (Thermo Fisher Scientific) and sent for bidirectional Sanger sequencing using an ABI 3730XL DNA Analyzer (1st BASE, Singapore). Sequences were checked for quality, assembled, and trimmed using BioEdit v7.2.5. The obtained DNA sequences were submitted to the NCBI GenBank with the accession numbers (PQ896696- PQ896705).

### Molecular diversity and demographic analysis

The edited *COI* sequences were aligned using ClustalW implemented in MEGA 11 [15]. A multiple sequence alignment was performed to compare the collected sequences with reference sequences from GenBank. Analysis of population differentiation through pairwise tests (φST) alongside population structure patterns by Analysis of Molecular Variance (AMOVA) was run in Arlequin version 3.5.1.3. The data analysis for haplotypes was conducted through DnaSp ver. 5 program. A ND2 haplotype network was created through the median-joining (MJ) approach [16] with POPART [17].

### Phylogenetic analysis

Phylogenetic relationships were inferred using the maximum likelihood (ML) and Bayesian inference (BI) methods. The ML tree was constructed in IQ-TREE v2.1.3 [18] with the best-fit nucleotide substitution model determined by ModelFinder. Support for nodes was evaluated using 1000 bootstrap replicates. The BI analysis was performed in BEAST v1.10.4 [19] with a Yule speciation model [20] and Markov Chain Monte Carlo (MCMC) sampling for 10 million generations. The first 25% of trees were discarded as burn-in, and posterior probabilities were estimated from the remaining trees. Genetic distances were computed using the p-distance model in MEGA 11. The outgroup sequence used for phylogenetic analysis was *Agama agama* (voucher CIBIO.983), a taxonomically related species from the family Agamidae. The final phylogenetic tree was visualized using FigTree v1.4.4.

## Results

### Genetic distance analysis

The pairwise genetic distances were analysed by the p-distance model in MEGA 11 software. The genetic distance analysis revealed clear patterns of evolutionary divergence among the 10 analyzed samples. The genetic distances in Table 1 presented as percentage values, shows the degree of genetic separation between the samples, while the upper triangular values show the standard error associated with each pairwise comparison. The results indicated two distinct genetic lineages within the dataset. The genetic analysis of samples IS.1 to IS.6 produced very small genetic distances (between 0.0% and 1.0%) which demonstrates these belongs to the same species (*Uromastyx*). The samples show low evolutionary divergence which support their genetic classification as a single genetic cluster.

**Table 1:** Genetic distance estimates among *Uromastyx* and *Calotes* samples based on evolutionary divergence.

Specimens Code/accession No	1	2	3	4	5	6	7	8	9	10
IS.1_PQ896696		0.01	0.01	0.00	0.01	0.01	0.25	0.30	0.25	0.30
IS.2_PQ896697	0.01		0.00	0.01	0.00	0.01	0.27	0.28	0.27	0.28
IS.3_PQ896698	0.01	0.00		0.01	0.00	0.01	0.26	0.28	0.26	0.28
IS.4_PQ896699	0.00	0.01	0.01		0.01	0.00	0.24	0.27	0.24	0.27
IS.5_PQ896700	0.01	0.00	0.00	0.01		0.01	0.26	0.28	0.26	0.28
IS.6_PQ896701	0.01	0.01	0.01	0.00	0.01		0.26	0.28	0.26	0.28
IS.7_PQ896702	0.25	0.27	0.26	0.24	0.26	0.26		0.14	0.00	0.14
IS.8_PQ896703	0.30	0.28	0.28	0.27	0.28	0.28	0.14		0.14	0.00
IS.9_PQ896704	0.25	0.27	0.26	0.24	0.26	0.26	0.00	0.14		0.14
IS.10_PQ896705	0.30	0.28	0.28	0.27	0.28	0.28	0.14	0.00	0.14	

On the other hand, samples IS.7 to IS.10 form a separate lineage, with intra-group genetic distances of 0.0% to 14.0%, indicating a closer evolutionary relationship among them. However, their genetic distances from samples IS.1 to IS.6 are significantly higher, ranging from 24.0% to 30.0%, confirming their classification as a distinct species. Results from samples IS.7 and IS.9 indicate 0.0% genetic distance which proves their exact genetic identity supporting their species placement. The genetic distance value of 0.0% from both samples IS.8 and IS.10 verifies that these two specimens belong to the same species (*Calotes*). The genetic separation between these two clusters aligns with known taxonomic distinctions between *Calotes* and *Uromastyx*. The obtained DNA sequences of these 10 samples were submitted to the NCBI GenBank, with the following accession numbers: PQ896696 to PQ896705, providing a valuable reference for future genetic and evolutionary studies.

### Genetic diversity of *Calotes* and *Uromastyx* species

Genetic analysis of *Calotes* and *Uromastyx* species based on partial *COI* sequences revealed significant levels of genetic diversity, gene flow, and population differentiation using DnaSP analysis. A total of 10 sequences were analyzed, comprising six from *Uromastyx* and four from *Calotes*. DnaSP analysis identified a total of 5 haplotypes and 46 number of segregating sites. The *Uromastyx* population exhibited a lower number of segregating sites (S = 2) and nucleotide diversity (Pi = 0.00815) compared to *Calotes* (S = 21, Pi = 0.10072). Haplotype diversity was slightly higher in *Uromastyx* (Hd = 0.73333) than in *Calotes* (Hd = 0.66667), but the average number of nucleotide differences was considerably greater in *Calotes* (K = 14.000) than in *Uromastyx* (K = 1.13333), indicating greater genetic variation within the *Calotes* population. The total dataset revealed 46 segregating sites, five haplotypes, and an overall nucleotide diversity (PiT) of 0.16643. Genetic differentiation estimates showed a significant chi-square value (Chi² = 10.000, *P* = 0.0404), suggesting moderate differentiation between the two populations. The fixation index (*Fst*) was 0.80681, indicating a high level of genetic differentiation, with low gene flow (Nm = 0.12) between the populations. Additional genetic divergence metrics, including Nei’s Gst (0.17214) and Hudson’s Snn (1.00000), further supported strong differentiation. The pairwise genetic distance (*Dxy* = 0.28177) and net nucleotide divergence (*Da* = 0.22734) suggested substantial genetic separation between *Calotes* and *Uromastyx* within the Cholistan Desert.

Pairwise comparisons indicated an average of 72.511 nucleotide differences across 430.71 analyzed sites, with an overall nucleotide diversity (*Pi*) of 0.15823. At the individual site level, 676 positions were analyzed, identifying 195 polymorphic sites and an average of 104.946 differences, with a *Pi* value of 0.15525. Within *Uromastyx*, the number of polymorphic sites was low (S = 2), with an average nucleotide difference (*k*) of 1.133 and nucleotide diversity (*Pi*) of 0.00815. In contrast, *Calotes* exhibited a higher level of polymorphism (S = 21), with *k* = 14.000 and *Pi* = 0.10072, suggesting greater genetic variation. The total dataset showed 46 polymorphic sites and 55 mutations, with an average nucleotide difference (*k*) of 23.133 and nucleotide diversity (*PiT*) of 0.16643. Between populations, 32 fixed differences were observed, with 21 mutations polymorphic in *Calotes* but monomorphic in *Uromastyx*, and two mutations polymorphic in *Uromastyx* but monomorphic in *Calotes*. The genetic divergence between populations was substantial, with an average nucleotide difference of 39.167 and nucleotide substitution per site (*Dxy*) of 0.28177, increasing to 0.35387 with Jukes and Cantor correction. The net nucleotide substitution per site (*Da*) was 0.22734, reflecting significant evolutionary separation. Pairwise analysis further supported these findings, with *k* = 23.133, an observed variance of 333.4364, and a coefficient of variation (C.V.) of 0.8091. Ramos-Onsins and Rozas’ R2 statistic (0.2514) suggested demographic influences on genetic variation.

### Demographic analyses

Demographic analyses based on Fu’s *Fs*, Tajima’s *D*, and other neutrality tests provided insights into the evolutionary history and population dynamics of *Calotes* and *Uromastyx* species in the Cholistan Desert. A total of 10 sequences were analyzed, covering 683 nucleotide sites, with 139 sites remaining after excluding alignment gaps. The number of polymorphic (segregating) sites was 46, with 55 total mutations. The average number of pairwise nucleotide differences (*k*) was 23.133, and nucleotide diversity (*Pi*) was 0.16643. Haplotype analysis revealed five distinct haplotypes, with a haplotype diversity (*Hd*) of 0.867 and a variance of 0.00509. Fu’s *Fs* statistic was 7.007, and Strobeck’s *S* statistic was 0.008, suggesting moderate genetic variation within the populations. Fu and Li’s *D* and *F* test statistics were 1.61114 and 1.66242, respectively, while Achaz’s *Y* statistic was 0.39171. Statistical significance was detected for Fu and Li’s *D* (*P* < 0.02) and *F* (*P* < 0.05), indicating potential deviations from neutrality, possibly due to population expansion or purifying selection. Tajima’s *D* statistic was 0.93030 (*P* > 0.10), indicating no significant departure from neutrality, suggesting a stable population size. The analyses showed no significant evidence of selection pressure, and the presence of multiple haplotypes with moderate genetic diversity suggests a relatively stable but slightly structured population.

The descriptive statistics in Table 2 show the genetic variation in *Calotes* and *Uromastyx* revealing moderate genetic diversity within the analyzed *COI* sequences. The haplotype diversity (Hd = 0.867) indicates a relatively high level of genetic variation among individuals. Nucleotide diversity (π = 0.16643) and the Watterson estimator (θw = 0.13987) suggest considerable polymorphism, with 46 segregating sites (S) identified. The average number of nucleotide differences (Π = 23.133) further supports the presence of genetic differentiation. Tajima’s D (D = 0.93030) was not statistically significant, indicating that the population is likely evolving neutrally without strong selection pressures. However, Fu’s FS test (FS = 7.007) suggests a deviation from neutrality, possibly due to demographic expansion or selection. The Ramos-Onsins and Rozas R2 statistic (R2 = 0.2514) and raggedness index (r = 0.1057) also indicate potential population structure changes. The estimated expansion parameter (τ = 5.518) and expected expansion time (texp = 17.615) further suggest that these populations may have undergone past demographic changes.

**Table 2:** Descriptive Statistics on Genetic Variation in *Calotes* and *Uromastyx.*

Gene	n	Hd	π	θw	S	h	Π	Tajima’s D	FS	R2	r	τ	texp
*COI*	10	0.867	0.16643	0.13987	46	5	23.133	0.93030	7.007	0.2514	0.1057	5.518	17.615

n—Number of sequences analyzed. Hd—Haplotype diversity [Nei, 1978]. π—Nucleotide diversity. θw—Watterson estimator. S—Number of segregating sites. h—Number of haplotypes. Π—Average number of nucleotide differences between sequence pairs [Nei, 1978]. D—Tajima’s neutrality test [Tajima, 1989]. FS—Fu’s neutrality test [Fu, 1997]. R2—Ramos-Onsins and Rozas neutrality test (Ramos- Onsins and Rozas, 2002]. r—Raggedness statistic [Harpending, 1994]. τ—Tau for populations indicated to deviate from demographic equilibrium. texp—Expected time since population expansion.

### Population genetic structure analysis

In Table 3, AMOVA results revealed significant genetic differentiation between *Uromastyx* and *Calotes* populations. The variance among populations accounted for 40.42% of the total genetic variation, while the remaining 59.58% was attributed to within-population diversity. The fixation index (FST = 0.40421) suggests moderate to high genetic differentiation between the two species, indicating restricted gene flow and potential reproductive isolation. The highly significant P-value (0.00880, P < 0.01) were obtained from permutation tests confirms that the observed genetic structure is unlikely to have arisen by chance.

**Table 3:** Analysis of Molecular Variance (AMOVA) Results.

Source of Variation	df	Sum of Squares	Variance Components	Percentage of Variation (%)
Among populations	1	422.200	67.29427	40.42
Within populations	8	793.500	99.18750	59.58
Total	9	1215.700	166.48177	100.00

The molecular diversity indices indicate substantial differences between *Uromastyx* and *Calotes*. The number of substitutions was significantly higher in *Calotes* (85 substitutions) compared to *Uromastyx* (2 substitutions), highlighting greater genetic diversity within *Calotes*. Similarly, *Calotes* exhibited a much larger number of private substitution sites (84), reinforcing its higher intraspecific variability. The nucleotide diversity (Pi) was also considerably higher in *Uromastyx* (250.600) than in *Calotes* (111.333), suggesting greater genetic variation within *Uromastyx*. However, *Calotes* exhibited a higher Theta_S (46.36) compared to *Uromastyx* (0.87), indicating a larger historical population size or a recent population expansion in *Calotes*.

**Fig 1:**
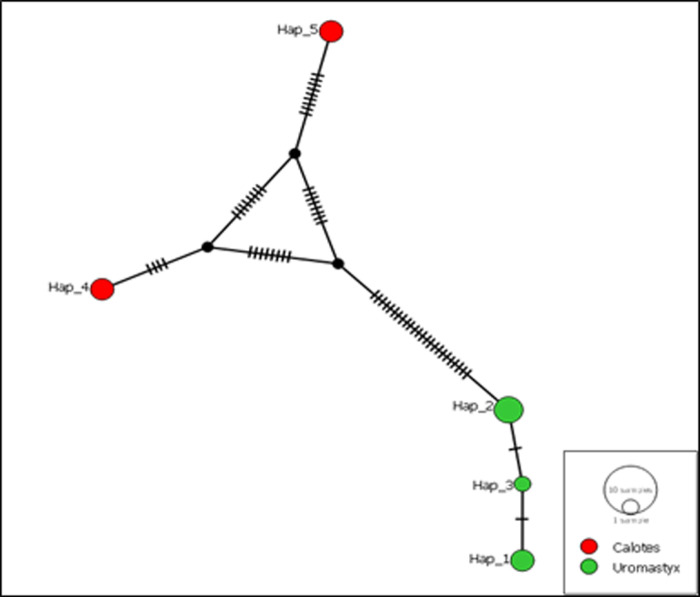
ND2 median-joining network of *Clotes* and *Uromastyx.*

The ND2 haplotype network (Fig 1), constructed using the median-joining (MJ) approach in POPART, reveals clear genetic separation between *Calotes* (red) and *Uromastyx* (green), with no shared haplotypes. *Calotes* exhibits a more interconnected network, suggesting recent diversification and possible gene flow, while *Uromastyx* shows more dispersed haplotypes, indicating a historically structured population with limited genetic exchange. The greater mutational divergence in *Uromastyx* suggests longer evolutionary separation, whereas *Calotes* appears to have undergone recent genetic diversification, likely due to environmental adaptation or population expansion. The distinct clustering of haplotypes supports strong genetic isolation between the species, reinforcing their classification as separate evolutionary units.

### Results of phylogenetic analysis

**Fig 2:**
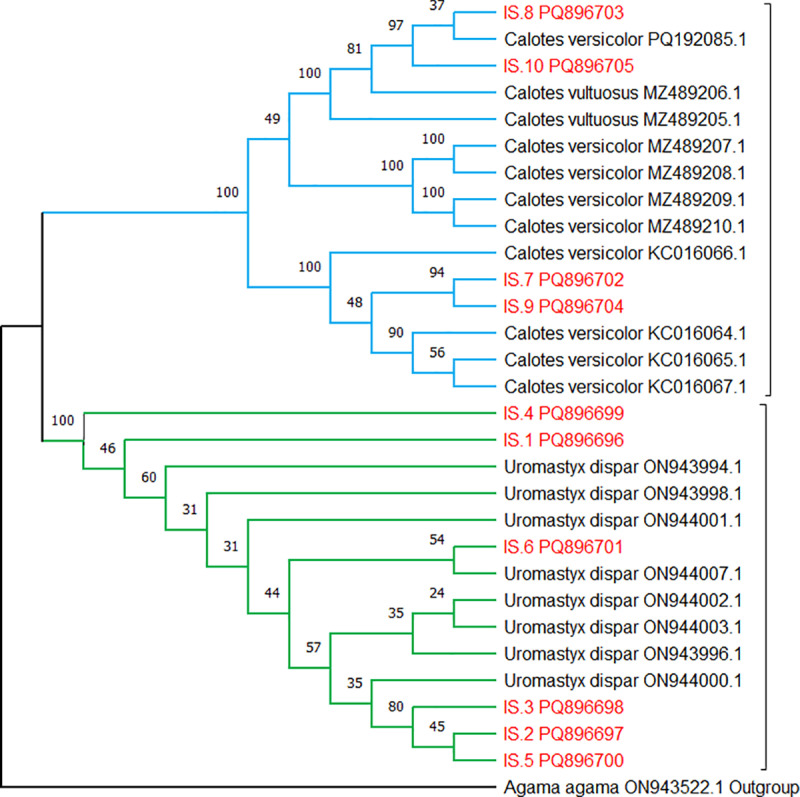
Phylogenetic tree of *Calotes* and *Uromastyx* species based on Neighbor-Joining method using Maximum Composite Likelihood analysis.

The phylogenetic relationships among the analyzed taxa were inferred using the Neighbor-Joining (NJ) method with the Maximum Composite Likelihood approach (Fig 2). The resulting phylogenetic tree presents two well-supported major clades, corresponding to the *Calotes* (blue) and *Uromastyx* (green) species, with *Agama agama* serving as an outgroup to root the tree.

The *Calotes* clade groups together samples *IS.7 PQ896702*, *IS.8 PQ896703*, *IS.9 PQ896704*, and *IS.10 PQ896705*, along with reference sequences of *Calotes versicolor* and *Calotes vultuosus*. The close association of study samples with *C. versicolor* suggests their genetic affinity with this species. The *Uromastyx* clade contains samples *IS.1 PQ896696*, *IS.2 PQ896697*, *IS.3 PQ896698*, *IS.4 PQ896699*, *IS.5 PQ896700*, and *IS.6 PQ896701*, clustering with reference sequences of *Uromastyx dispar*. The genetic structure within this clade suggests a degree of variation among the *Uromastyx* samples, likely due to population-level differentiation.

**Fig 3:**
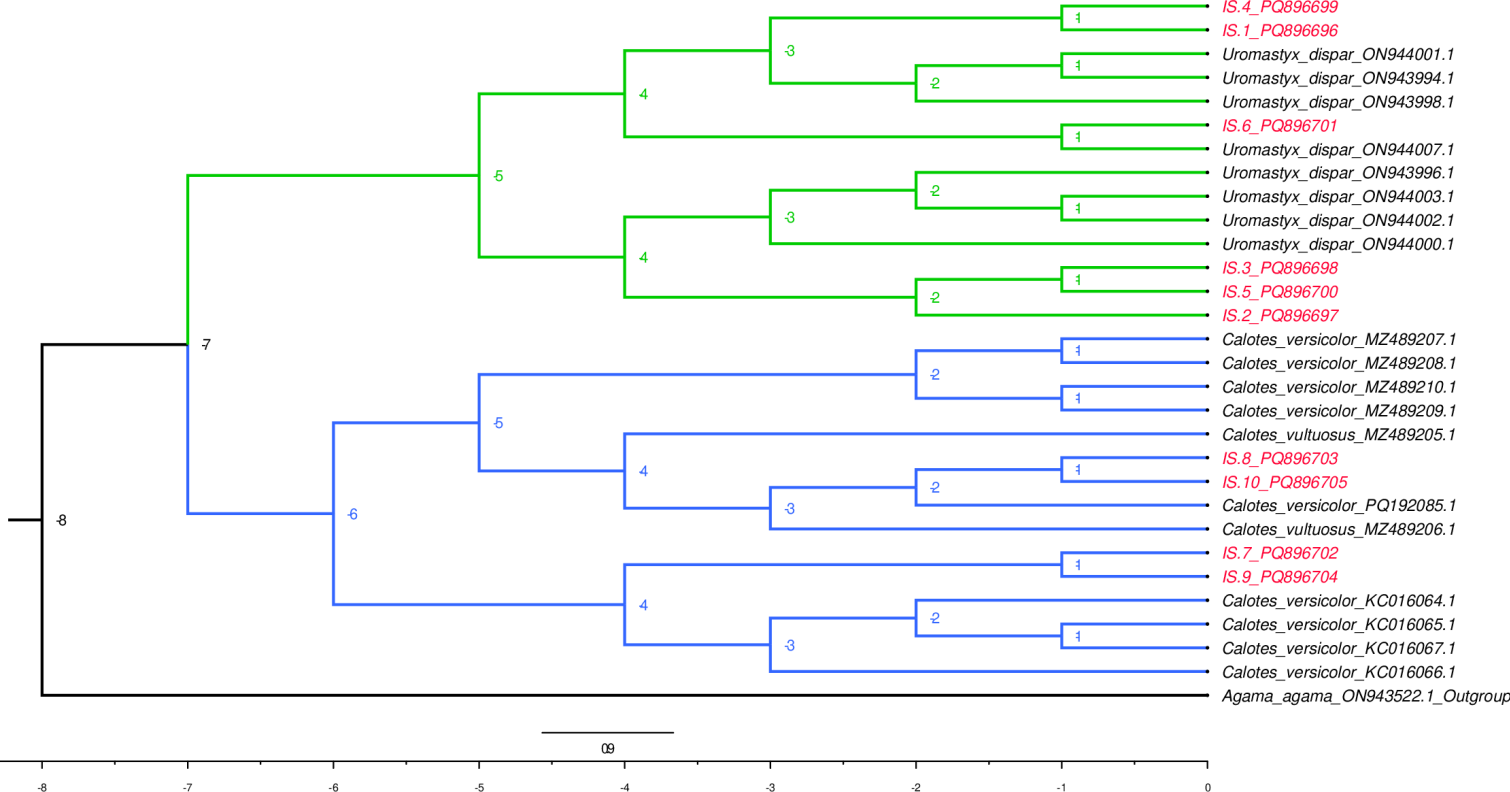
The phylogenetic relationships of *Calotes* and *Uromastyx*: Bayesian Inference with BEAST.

The phylogenic tree for the species of *Calotes* and *Uromastyx* were established with Bayesian Evolutionary Analysis through BEAST software with the help of Fig Tree v1.4 (Fig 3). The analysis used the General Time Reversible (GTR) model and performed the strict clock approach to calculate divergence time and posterior probabilities of each node. The phylogenetic tree was rooted to *Agama agama* outgroup as a measure of obtaining the evolutionary basal line. The posterior probability values are given above the branches signifying the measure of the clade support. The tree topology distinctly separates two major clades: one comprising *Calotes versicolor* and *Calotes vultuosus*, and the other containing *Uromastyx dispar* sequences. The high posterior probability values at key nodes support the reliability of these clades. Within the *Calotes* clade, multiple accessions of *C. versicolor* cluster closely, indicating low genetic divergence among them. Conversely, the *Uromastyx* clade exhibits slightly higher divergence, suggesting greater genetic diversity within the species. Additionally, the inclusion of samples IS.1_PQ896696 to IS.10_PQ896705 within these clades demonstrates their taxonomic affiliation. The samples IS.7_PQ896702, IS.3_PQ896698, and IS.5_PQ896700 cluster within the *Uromastyx* lineage, while IS.10_PQ896705, IS.9_PQ896704, and IS.8_PQ896703 are firmly placed within the *Calotes* clade. This suggests that the analyzed specimens are genetically aligned with their respective reference sequences. The phylogenetic reconstruction also highlights genetic divergence patterns, indicating that *Uromastyx* has undergone more significant evolutionary changes compared to *Calotes*. The findings provide strong molecular evidence for the distinct evolutionary pathways of these genera, reinforcing their taxonomic classification.

## Discussion

The present study provides the first comprehensive assessment of genetic diversity and phylogenetic relationships of *Calotes* and *Uromastyx* lizards in the Cholistan Desert, Pakistan, based on mitochondrial *COI* gene sequences. Our results revealed significant genetic divergence between these genera, with distinct evolutionary lineages. We contextualize these results in light of existing literature, emphasizing their implications for conservation and species delineation.

The analysis of *COI* sequences demonstrated notable genetic differentiation between *Calotes* and *Uromastyx*, with pairwise distances ranging from 24% to 30%, consistent with their taxonomic separation at the genus level. Within *Uromastyx*, low intraspecific divergence (0.0–1.0%) suggests a closely related group, likely representing *U. hardwickii*, the only species documented in this region. This finding corroborates earlier studies that reported minimal mitochondrial divergence among *Uromastyx* populations in arid zones [8,21,22]. However, the higher nucleotide diversity in *Calotes* (π = 0.10072 vs. *Uromastyx*: π = 0.00815) indicates greater genetic variability, possibly reflecting adaptive responses to heterogeneous habitats or historical population expansions. Similar patterns were observed in *Calotes versicolor* populations across South Asia, where environmental gradients drove localized genetic structuring [5].

In this study, the AMOVA results further emphasized this divergence, with 40.42% of genetic variation attributed to differences between genera (*FST* = 0.404). Such pronounced differentiation aligns with studies on other agamid lizards, where desert-dwelling species exhibit stronger population structure due to habitat fragmentation [9,23]. Restricted gene flow (*Nm* = 0.12) between *Calotes* and *Uromastyx* likely stems from ecological niche partitioning—*Uromastyx* inhabits arid scrublands, while *Calotes* occupies vegetated areas—a phenomenon documented in sympatric agamids [24].

The ML and BI phylogenies resolved *Calotes* and *Uromastyx* as monophyletic clades, with *Agama agama* as a robust outgroup. Within *Calotes*, the clustering of Cholistan samples with *C. versicolor* (bootstrap >90%) supports their identification as members of this widespread species complex. However, the high intraspecific divergence (up to 14%) in our samples raises questions about potential cryptic diversity. Comparable findings were reported by Maulana, Pakpahan [12] in Indonesian *Calotes*, where *COI*-based analyses revealed unresolved species complexes. While mitochondrial markers alone cannot conclusively delineate species, our results echo calls for integrative taxonomic approaches combining morphology and nuclear genes (e.g., *RAG1*) to clarify these boundaries [14].

In our study, the low genetic distances among Cholistan specimens (for *Uromastyx*) contrast with the deep divergences observed between Saharo-Arabian congeners [21]. This uniformity may reflect recent colonization or bottleneck events, as proposed for *Saara hardwickii* in India [25]. Notably, the absence of shared haplotypes between genera in the median-joining network underscores their long-term evolutionary isolation, consistent with fossil evidence placing their divergence in the mid-Miocene [26]. Neutrality tests (Tajima’s *D* = 0.93030, *P* > 0.10; Fu’s *FS* = 7.007) suggested stable population sizes without recent bottlenecks. However, the raggedness index (*r* = 0.1057) and expansion parameter (τ = 5.518) imply past demographic fluctuations, possibly linked to Pleistocene climatic shifts that repeatedly altered desert habitats [9]. The higher haplotype diversity in *Uromastyx* (*Hd* = 0.733) compared to *Calotes* (*Hd* = 0.667) may reflect differential survival during aridification, as *Uromastyx*’s burrowing behavior buffers against environmental extremes [10].

These genetic patterns have urgent conservation implications. Both genera face habitat loss from agricultural expansion and overharvesting (*Uromastyx* for meat, *Calotes* for pet trade). The low genetic diversity in *Uromastyx* signals vulnerability to inbreeding, mirroring declines in North African populations [11]. Conversely, *Calotes*’ variability may confer resilience, but its reliance on fragmented vegetation necessitates habitat corridors. Regionally, our findings advocate for IUCN reassessments of *U. hardwickii*, currently listed as Least Concern, and underscore the Cholistan Desert as a biogeographic refugium worthy of protected status.

## Conclusion

This study represents the first comprehensive genetic assessment of *Calotes* and *Uromastyx* lizards in the Cholistan Desert, Pakistan, utilizing mitochondrial *COI* gene analysis to elucidate patterns of diversity, differentiation, and evolutionary relationships. Our results confirm the distinct phylogenetic separation between these genera, with significant genetic divergence (24–30%) that aligns with their established taxonomic classification. Within *Uromastyx*, low intraspecific variation suggests a relatively homogeneous population, likely corresponding to *U. hardwickii*, while the higher diversity observed in *Calotes* points to potential cryptic lineages or adaptations to local ecological conditions. The findings contribute to a growing body of evidence highlighting the utility of *COI* barcoding for preliminary species identification and conservation prioritization in understudied arid ecosystems. By providing baseline genetic data, this study not only advances our understanding of agamid lizard evolution in Cholistan Desert in South Punjab, Pakistan but also establishes a framework for ongoing biodiversity monitoring in the region. Further integrative research, combining morphological, ecological, and genomic approaches, will be essential to clarify species boundaries and inform effective management strategies for these taxa in one of Pakistan’s most fragile desert ecosystems.

## Supporting information

S1 File S1 raw images. (PDF)
