# Supplementary figures and images for: Genetic diversity and phylogenetic relationships of Calotes and Uromastyx in the Cholistan Desert, Pakistan, based on COI gene analysis
Source: PLoS One. 2025 Jun 17;20(6):e0324053. doi: 10.1371/journal.pone.0324053 (PMC12173366; doi:10.1371/journal.pone.0324053)

Raw Image

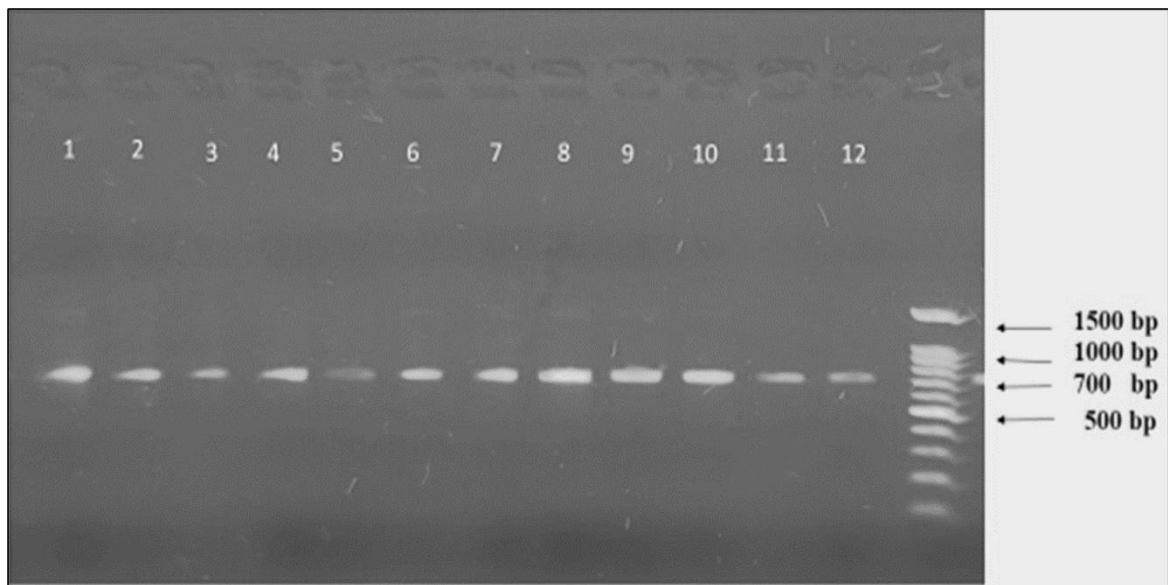

Original Image

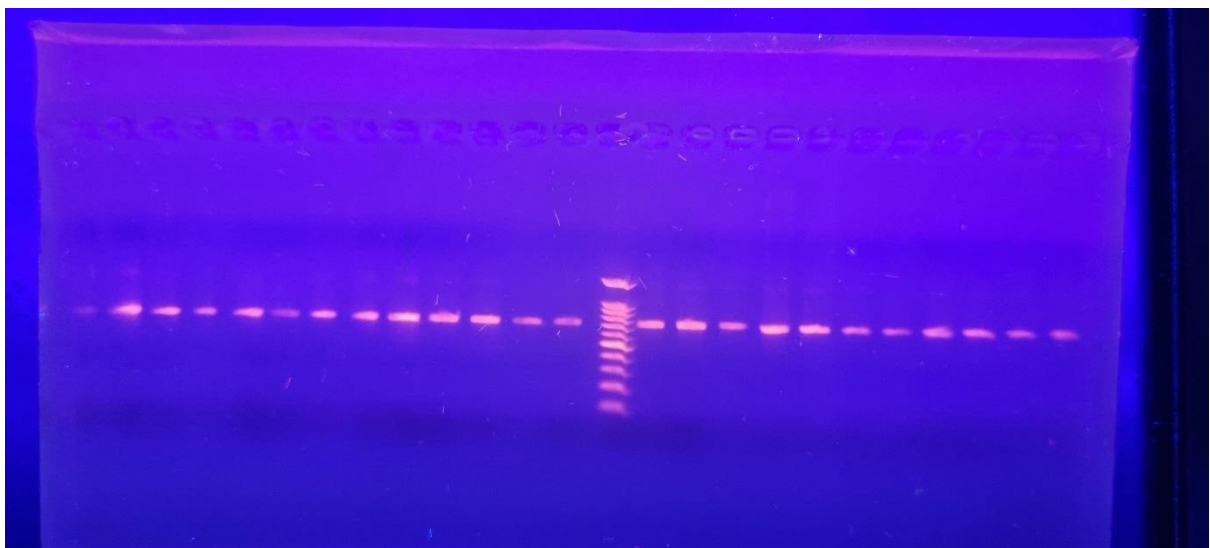

Supplement: S1 File — (PDF) [file pone.0324053.s001.pdf]
